# Identification of a functional DddX-like enzyme that catalyzes dimethylsulfoniopropionate cleavage in marine bacteria

**DOI:** 10.1016/j.engmic.2026.100296

**Published:** 2026-07-30

**Authors:** Shu-Yan Wang, Fei Xu, Meng Meng, Zeng-Tian Gu, Min Zhang, Yu-Zhong Zhang, Nan Zhang, Chun-Yang Li

**Affiliations:** a School of Bioengineering, Qilu University of Technology (Shandong Academy of Sciences), Jinan 250353, China; b Shandong Academy of Chinese Medicine, Jinan 250014, China; c Marine Biotechnology Research Center, State Key Laboratory of Microbial Technology, Shandong University, Qingdao 266237, China; d MOE Key Laboratory of Evolution and Marine Biodiversity, State Key Laboratory of Marine Food Processing and Safety Control, Frontiers Science Center for Deep Ocean Multispheres and Earth System, Shandong Provincial Key Laboratory of Microbial Resource Exploration and Innovative Utilization & College of Marine Life Sciences, Ocean University of China, Qingdao 266003, China; e Joint Research Center for Marine Microbial Science and Technology, Shandong University and Ocean University of China, Qingdao 266237, China; f Laboratory for Marine Biology and Biotechnology, Qingdao Marine Science and Technology Center & Laoshan Laboratory, Qingdao 266237, China

**Keywords:** Dimethylsulfoniopropionate lytic enzyme, Dimethyl sulfide, Marine *Roseobacter* group, Marine sulfur cycle

## Abstract

Dimethylsulfoniopropionate (DMSP), a ubiquitous organosulfur compound in marine environments, plays an important role in global sulfur cycling and climate regulation. Many marine microorganisms catabolize DMSP through DMSP lytic enzymes, producing the infochemical and potential climate-cooling gas dimethyl sulfide (DMS). The marine *Roseobacter* group (MRG) is one of the most abundant heterotrophic bacteria groups in marine ecosystems and an active participant in DMSP cycling. To expand our knowledge of the diversity of DMSP lytic enzymes, we identified and characterized a functional DddX-like enzyme from the MRG strain *Neptunicoccus cionae* H-12 and other related bacteria, such as *Leisingera* spp., *Sulfitobacter* spp., and *Roseovarius* spp. This enzyme catalyzes the cleavage of DMSP into DMS and acryloyl-CoA. Although the DddX-like enzyme belongs to the same acyl-CoA synthetase superfamily as DddX, a previously reported DMSP lytic enzyme, it shares low amino acid sequence identity (∼23%) with DddX. Phylogenetic analysis suggested that the DddX-like enzyme formed a separate clade from DddX, with the DddX-like enzyme predominantly distributed in the cosmopolitan MRG. Bioinformatic analysis indicated that the relative abundance of *dddX* and *dddX*-like genes, along with their transcripts, is notably high in marine environments, suggesting their important roles in DMSP cycling. Our findings enhance knowledge of DMSP lytic enzymes and provide novel insights into global sulfur cycling processes.

## Highlights

• A novel DddX-like DMSP lyase was identified from the marine *Roseobacter* group.
• The enzyme shares ∼23% identity with DddX and forms a distinct clade.
• The DddX-like enzyme catalyzes DMSP cleavage to DMS and acryloyl-CoA.
• The *dddX* and *dddX*-like genes and transcripts are abundant in marine environments.

## Graphical abstract

**Figure:**
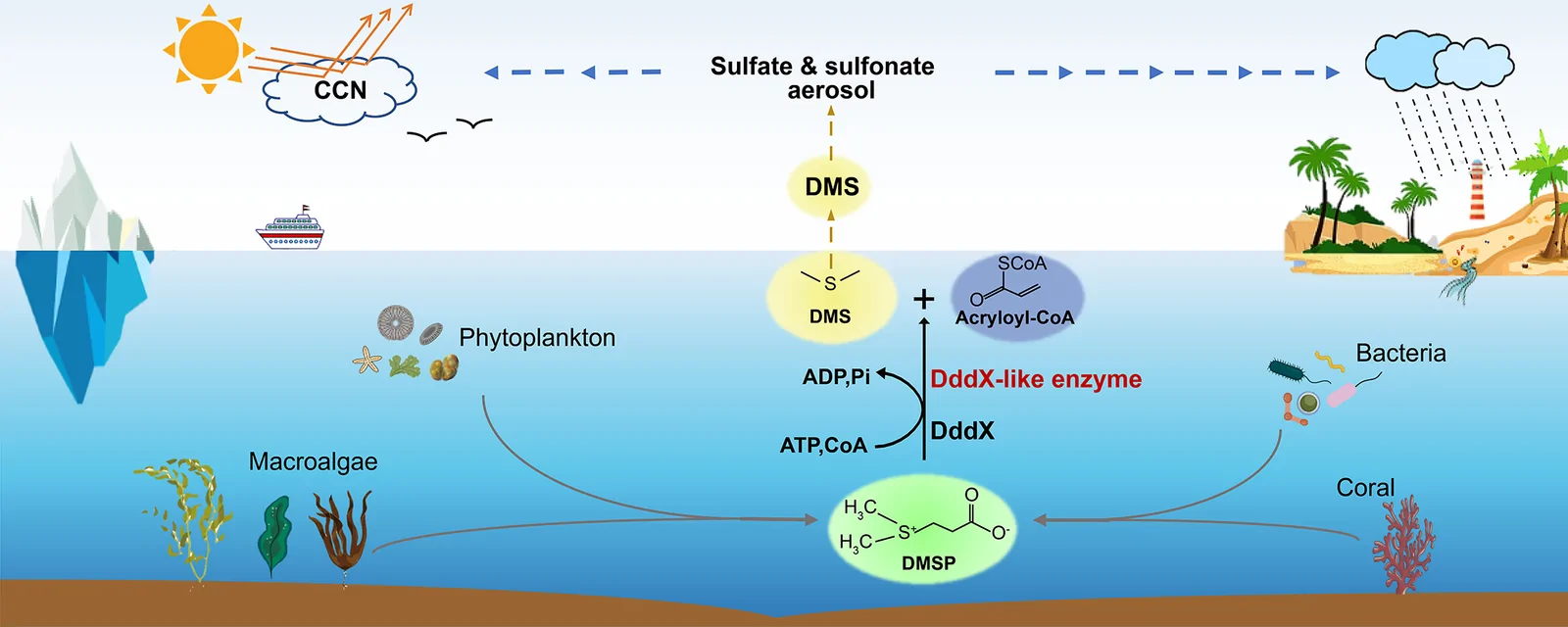

## 1 Introduction

Dimethylsulfoniopropionate (DMSP) is an organosulfur compound widely distributed across various marine environments, such as saltmarsh sediments, freshwater lakes, and coastal regions, and it is particularly prevalent in global oceans [[1], [2], [3], [4]]. It can act as an osmolyte, antioxidant, cryoprotectant, and predator deterrent, and it is also an important participant in global carbon and sulfur cycling [[5], [6], [7], [8], [9], [10]]. Diverse marine eukaryotes (including phytoplankton, macroalgae, and angiosperms) and some marine bacteria have been reported to produce DMSP [[11], [12], [13], [14]], and a large fraction of DMSP is released to marine environments following the grazing or senescence of DMSP producers [15]. Notably, marine bacteria, particularly the marine *Roseobacter* group (MRG) and the SAR11 clade, represent two major groups of heterotrophic bacteria in Earth’s surface oceans. These organisms can accumulate DMSP to high intracellular concentrations (millimolar) and catabolize it to produce volatile dimethyl sulfide (DMS) along with acrylate, 3-hydroxypropionate (3-HP)-CoA, or acryloyl-CoA [16]. DMS can diffuse out of the cell, and the acrylate/3-HP-CoA/acryloyl-CoA produced is further assimilated by downstream enzymes [16]. DMS is a potent signaling molecule for diverse organisms, including microorganisms, fish, marine mammals, and seabirds [9,[17], [18], [19], [20]]. Furthermore, in the atmosphere, DMS oxidation products contribute to cloud condensation nuclei formation, influencing the reflectivity of clouds and ultimately the global climate [[21], [22], [23]].

To date, 10 different DMSP lytic enzymes that convert DMSP to DMS have been identified (DddL, DddQ, DddW, DddY, DddK, DddU, DddP, Alma1, DddD, and DddX) spanning five phylogenetically distinct enzyme superfamilies [16]. Among them, DMSP lyases DddL, DddQ, DddW, DddY, DddK, and DddU all contain two conserved cupin motifs, and thus belong to the cupin superfamily [[24], [25], [26], [27], [28], [29]]. Meanwhile, DddP is the most prevalent bacterial DMSP lyase recorded in marine metagenomic datasets, and belongs to the M24 metallopeptidase family [30]. Finally, Alma1, which originates exclusively from eukaryotic algae, belongs to the aspartate racemase superfamily [31]. These eight DMSP lyases directly cleave the C–S bond of DMSP, yielding DMS and acrylate. In comparison, the DMSP-CoA transferase DddD, which belongs to the type Ⅲ acyl-CoA transferase family, requires acetyl-CoA as a cofactor to convert DMSP to DMS and 3-HP-CoA [32]. In contrast, the DMSP-CoA ligase DddX, a member of the acyl-CoA synthetase (ACD) superfamily, utilizes ATP, CoA, and Mg^2+^ as cofactors to catalyze the transformation of DMSP, producing DMS and acryloyl-CoA [33].

The MRG accounts for up to ∼25% of coastal marine bacterial communities, making it one of the most prevalent groups among marine bacteria [34]. *Roseobacter* species have been extensively documented for their ability to degrade DMSP [34]. For instance, the bacterium *Neptunicoccus cionae* H-12 (previously named *Amylibacter cionae* H-12) [35,36], which belongs to the MRG, catabolizes DMSP to generate DMS [29]. Notably, the deletion of one known DMSP lyase gene *dddU* in strain H-12 did not abolish its DMSP cleavage activity, indicating the existence of additional, yet unidentified, DMSP lytic enzyme(s) in this strain [29].

In this study, we used genetic and biochemical approaches to identify a functional DddX-like enzyme from *N. cionae* H-12. This enzyme converts DMSP to DMS and acryloyl-CoA. A bioinformatic analysis was conducted to investigate the distribution of the DddX-like enzyme among marine bacteria and to further assess the ecological significance of *dddX* and *dddX-*like genes in marine environments. The results provide novel insights into DMSP catabolism in marine ecosystems and will ultimately lead to a better understanding of the global sulfur cycling process.

## 2 Materials and methods

### 2.1 Bacterial strains and growth conditions

The *N. cionae* H-12 strain, along with its Δ*dddX*-like gene mutant and associated derivatives (Δ*dddX*-like gene/pHG101-*dddX*-like gene, Δ*dddX*-like gene/pHG101; Table S1), were cultivated in Marine Broth 2216 medium at 25°C and 180 rpm. Kanamycin (50 µg/mL) was added to maintain the plasmids in the complemented strains. *Escherichia coli* strains were cultured in Lysogeny Broth (LB) medium at 37°C and 180 rpm.

### 2.2 Genetic manipulations of *N. cionae* H-12

To generate an in-frame deletion mutant of the *dddX*-like gene, genomic DNA was isolated from strain H-12 using a commercial bacterial DNA extraction kit (BioTeke, China). The upstream and downstream regions of the *dddX*-like gene were amplified, fused, and subsequently ligated into the suicide vector pHGM01. The resulting recombinant plasmid was initially introduced into *E. coli* WM3064 via transformation, then transferred into the wild-type (WT) H-12 strain through conjugal transfer [37]. Following homologous recombination, the *dddX*-like gene deletion mutant was successfully isolated and verified.

For genetic complementation, the full-length *dddX*-like gene, inclusive of its native promoter, was amplified and then ligated into the plasmid pHG101. The resulting construct, along with the empty pHG101 vector as a control, was transformed into *E. coli* WM3064 cells. Following this, each plasmid was separately conjugated into the Δ*dddX*-like gene mutant strain. The successful introduction of the *dddX*-like gene was subsequently verified through colony PCR. All DNA fragments generated by PCR for cloning were verified via sequencing. The oligonucleotide primers utilized in this study are listed in Table S2.

### 2.3 Quantification of DMS using gas chromatography (GC)

To probe DMSP catabolism and DMS production, *N. cionae* H-12 and its derivative strains were cultured in Marine Broth 2216 medium until they reached the late-exponential growth phase. Bacterial cells were then collected via centrifugation, rinsed three times with sterile artificial seawater, and resuspended to an OD_600_ of 0.6. Aliquots of this cell suspension were transferred into gas-tight sealed bottles containing minimal medium supplemented with 1 mM DMSP. These vials were incubated overnight for 12 h at 25°C. Following incubation, the concentration of DMS accumulated in the headspace of the individual culture vials was determined quantitatively using a GC system (GC-2030, Shimadzu, Japan) equipped with a flame photometric detector. Chromatographic separation was achieved on a glass chromatography column (CP7529, 0.32 mm × 30 m × 4.00 µm; Agilent, USA) and maintained at an isothermal temperature of 100°C. Ultra-pure nitrogen served as the carrier gas, which was injected at a constant flow rate of 3 mL/min. The culture medium without bacterial inoculation served as the control group. To quantify DMS concentrations, a calibration curve was established using seven distinct concentrations of DMS standards (0.5–500 µM). The detection limit for headspace DMS detection was 0.5 nM. For normalization, the protein content of the bacterial samples was determined using a Pierce BCA Protein Assay Kit (Thermo, USA). All experiments were conducted on three independently prepared biological replicates to ensure statistical reliability.

### 2.4 Gene synthesis, point mutation, protein expression, and purification

The full-length sequence of the *dddX*-like gene and several of its homologs were subjected to codon optimization to ensure efficient heterologous expression in *E. coli* BL21(DE3) cells. The corresponding genes were chemically synthesized using the solid-phase phosphoramidite method and subsequently inserted into the pET-22b expression vector (Sangon Biotech Co., Ltd., China). In addition to serving as the expression plasmid, this recombinant construct was also used as the DNA template for PCR-based site-directed mutagenesis. The oligonucleotide primers employed are listed in Table S2. To ensure heterologous expression, each of the constructed plasmids was separately overexpressed in *E. coli* BL21(DE3) cells and cultured in ampicillin (100 µg/mL)-containing LB medium. Upon reaching the early stationary phase, isopropyl-β-D-thiogalactopyranoside (0.5 mM) was added to the medium, and incubation was continued at a reduced temperature of 18°C for 16 h. Following induction, bacterial cells were first collected via centrifugation and then resuspended in a lysis buffer consisting of 50 mM Tris-HCl (pH 8.0), 100 mM NaCl, and 0.5% glycerol. Cell disruption was achieved using a cryogenic pressure crusher. The resulting crude lysate was clarified by centrifugation and subsequently subjected to purification in a Ni²⁺-nitrilotriacetic acid column (Cytiva, USA). Unbound proteins were first removed using a wash buffer (15 mM imidazole, 50 mM Tris-HCl, 100 mM NaCl, and 0.5% glycerol), and the target protein was subsequently eluted with an elution buffer (250 mM imidazole, 50 mM Tris-HCl, 100 mM NaCl, and 0.5% glycerol). The eluted fractions containing the target protein were further purified via gel filtration in a Superdex™ G200 column (Cytiva, USA) with a buffer containing 10 mM Tris-HCl (pH 8.0) and 100 mM NaCl. The standards used in gel filtration analysis were purchased from Cytiva, USA and included thyroglobulin (669 kDa), ferritin (440 kDa), aldolase (158 kDa), conalbumin (75 kDa), carbonic anhydrase (29 kDa), ribonuclease A (13.7 kDa), and aprotinin (6.5 kDa).

### 2.5 Product identification, enzyme assay, and characterization

The catalytic activity of the purified DddX-like enzyme was evaluated using a defined reaction system. The assay mixture, with a total volume of 200 µL, was composed of 100 mM Tris-HCl buffer (pH 8.0), 1 mM DMSP, 1 mM ATP, 1 mM CoA, and 2 mM MgCl_2_. This mixture was incubated at 40°C for 15 min, after which DMS production was analyzed via GC, and acryloyl-CoA was detected using liquid chromatography-mass spectrometry (LC-MS). In brief, the components in the mixture were separated using a high-performance liquid chromatography (HPLC) system (Dionex, USA) equipped with a SunFire C18 column (Waters, Ireland). Elution was conducted over a 24-min linear gradient, increasing from 1% to 20% (v/v) acetonitrile in 50 mM ammonium acetate buffer (pH 5.5). The effluent from the HPLC system was introduced into an impact HD mass spectrometer (Bruker, Germany) to determine the accurate mass-to-charge ratio (*m*/*z*) of the eluted compounds, and the eluent was continuously detected by monitoring ultraviolet absorbance at 260 nm. The ion species detected was [M+H]⁺. The reaction mixture with the same composition but without the DddX-like enzyme was used as the negative control. To ensure the reliability and reproducibility of the results, all assays were conducted using three independently prepared biological replicates.

To investigate the influence of temperature on the catalytic efficiency of the DddX-like enzyme, a series of activity assays were conducted across a broad thermal range. The standard reaction mixtures were incubated at various temperatures (0, 10, 20, 30, 40, 50, and 60°C) for 15 min. The effect of pH on the catalytic activity of the DddX-like enzyme was also evaluated. These assays were performed at the optimal temperature of 40°C, utilizing a universal Britton–Robinson buffer system to maintain pH values ranging from 5 to 11. The kinetic parameters of the DddX-like enzyme were determined by incubating the enzyme itself (0.1 µM) with varying DMSP concentrations (0.5, 0.8, 1, 1.5, 2, 5, and 8 mM) in a pH 8.0 buffer at 40°C for 15 min, and the kinetic constants were calculated by applying nonlinear analysis. Three independent biological replicates were analyzed for each sample.

### 2.6 Structure prediction

For the structural prediction, the full-length amino acid sequence of the DddX-like enzyme was uploaded to the AlphaFold Server web interface (https://alphafoldserver.com) using the default parameters, with the number of copies set to 1. The model with the highest-ranking score was selected for subsequent analysis. Structural alignments were performed and figures created using PyMOL (https://www.pymol.org/).

### 2.7 Circular dichroism (CD) spectroscopy assays

Circular dichroism (CD) spectroscopy was employed to analyze the secondary structure differences of the WT DddX-like enzyme and its mutant variants. All measurements were performed on a JASCO J-1500 spectropolarimeter (Japan) at room temperature (25°C), with a cell path length of 0.1 cm. Briefly, protein samples were diluted to a final concentration of 15 µM using a buffer consisting of 10 mM Tris-HCl (pH 8.0) and 100 mM NaCl. Spectral data were collected over the wavelength range of 200–260 nm, employing a scanning rate of 200 nm/min. The data reported are the mean of three technical replicates per sample.

### 2.8 Bioinformatics analyses

Homology searches were performed using BLASTP on the NCBI web server, using the DddX-like enzyme from *N. cionae* H-12 as the query against the RefSeq database (https://www.ncbi.nlm.nih.gov/refseq/). Sequences with >90% coverage and >60% identity were retained. These relatively high‑stringency settings were necessary to exclude other acetate‑CoA ligase family proteins that are unlikely to be involved in DMSP catabolism. From this filtered dataset, a total of 27 homologous sequences derived from bacterial strains with publicly available complete genome assemblies were chosen for subsequent phylogenetic analysis. The sequences were aligned using ClustalW software, and the evolutionary distances of the phylogenetic tree were estimated using the Poisson model. Bootstrap analyses were performed based on 1000 replications. Phylogenetic trees were constructed using the neighbor-joining method in MEGA 7 [38].

The distribution of DMSP lytic enzyme homologs in marine environments was analyzed as previously described [39]. Briefly, Hidden Markov Models (HMMs) were built using biochemically characterized protein sequences. As the retrieved DddX and DddX-like enzyme homologs were often fragmented [39], sequences with <60% coverage of the query were filtered out. Metagenomic and metatranscriptomic analyses were conducted using the *Tara Oceans* database with a cut-off value of ≤1e^-30^ (https://tara-oceans.mio.osupytheas.fr/ocean-gene-atlas/). The relative abundances of genes were normalized as the percent of mapped reads.

## 3 Results and discussion

### 3.1 Identification of a DddX-like enzyme

It has been reported that *Neptunicoccus cionae* H-12, isolated from the sea squirt *Ciona savignyi* [35], cleaves DMSP to DMS [29]. However, after targeted deletion of the known DMSP lyase gene *dddU* in H-12, the Δ*dddU* mutant still retained residual DMSP lyase activity, suggesting that this strain may possess unrecognized DMSP lytic enzyme(s) [29]. A genomic analysis of the H-12 strain was thus conducted to identify the novel DMSP lyase(s), and a protein of the ACD superfamily (WP_188673365.1), which shares ∼23% amino acid sequence identity to the DMSP lytic enzyme DddX, was found. We therefore termed this protein the DddX-like enzyme, and this phrase is used hereafter.

**Fig. 1:**
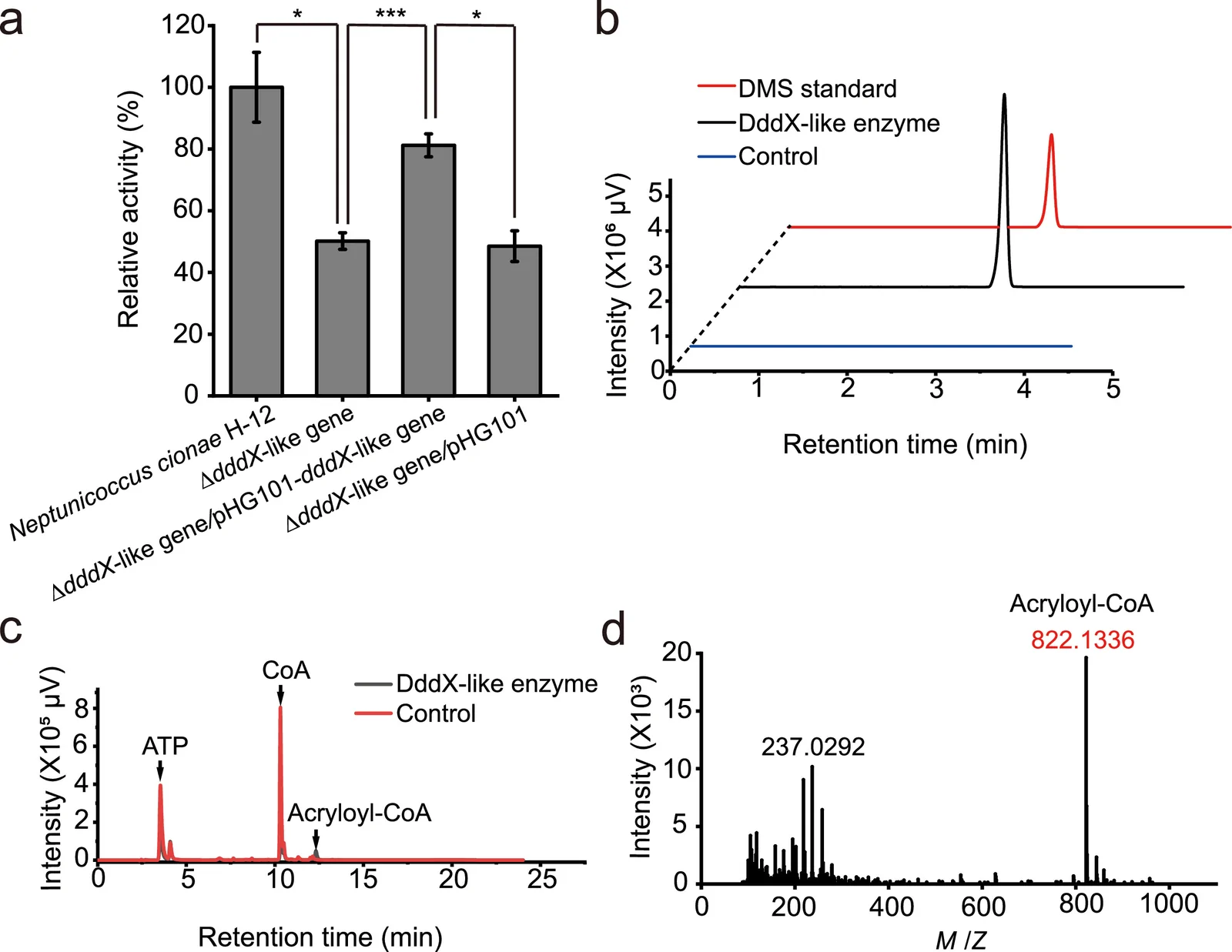
**Functional verification of the DddX-like enzyme.** (a) Phenotypic analysis of the WT strain *Neptunicoccus cionae* H-12, the Δ*dddX*-like gene mutant, the complemented mutant (Δ*dddX*-like gene/pHG101-*dddX*-like gene), and the mutant containing an empty vector (Δ*dddX*-like gene/pHG101). The error bars represent standard deviation of three independent biological replicates. *p < 0.05, ***p < 0.001 (two-tailed Student's *t*–test). (b) GC detection of DMS generated from DMSP cleavage by the recombinant DddX-like enzyme. (c) HPLC analysis of the recombinant DddX-like enzyme toward DMSP. (d) LC-MS analysis of the product acryloyl-CoA.

To validate the *in vivo* function of the *dddX*-like gene in strain H-12, a Δ*dddX*-like gene mutant was constructed via homologous recombination, in which the majority of the *dddX*-like gene was deleted. The GC analysis showed that the Δ*dddX*-like gene mutant strain produced ∼50% less DMS than the WT strain H-12. DMSP lyase activity was largely restored in the complemented strain carrying the *dddX*-like gene on the plasmid pHG101 (Fig. 1a). These data suggest that the *dddX*-like gene encodes a functional DMSP lytic enzyme responsible for producing DMS from DMSP *in vivo*. The contributions to DMSP degradation in strain H-12 from the DddX-like enzyme and DddU were nearly equal (Fig. S1). In addition, the double Δ*dddU*Δ*dddX*-like gene-knockout mutant retained some ability to cleave DMSP and produce DMS (Fig. S1), indicating the presence of additional unknown DMSP lytic enzyme(s) in H-12, which warrants further investigation. The coexistence of multiple DMSP lytic enzymes within a single bacterial strain is common in the MRG. For example, *Ruegeria pomeroyi* DSS-3 harbors three distinct DMSP lyases, DddP, DddQ and DddW, while *Dinoroseobacter shibae* DFL 12 contains DddL and DddD [15]. The possession of multiple DMSP lytic enzymes by a single strain likely reflects an adaptive strategy that enhances the ecological adaptations of microorganisms in dynamic marine environments.

To probe the enzymatic activity of the DddX-like enzyme *in vitro*, the full-length *dddX*-like gene of strain H-12 was heterologously overexpressed, and the recombinant DddX-like protein was purified. Gel filtration analysis indicated that the DddX-like protein maintains a dimeric structure in solution (Fig. S2). Given that enzymes of the ACD superfamily require CoA, ATP, and Mg^2+^ as essential cofactors for their catalytic activity [40,41], these three components were added into the reaction mixture to measure the enzymatic activity of the purified DddX-like enzyme. GC analysis indicated that the DddX-like enzyme catalyzed the release of DMS from DMSP (Fig. 1b), while HPLC and LC-MS analyses indicated that the DddX-like enzyme acted on DMSP to produce acryloyl-CoA (*m*/*z* = 822.1336) (Fig. 1c, d). These results confirm that the DddX-like enzyme is a functional DMSP lytic enzyme *in vitro* that can catalyze the degradation of DMSP to produce DMS and acryloyl-CoA, similar to DddX [33]. The removal of CoA, ATP, or Mg^2+^ abolished the enzymatic activity of the DddX-like enzyme (Fig. S3), suggesting that its enzymatic activity is dependent on ATP, CoA, and Mg^2+^.

### 3.2 Characterization of DddX-like enzyme

**Fig. 2:**
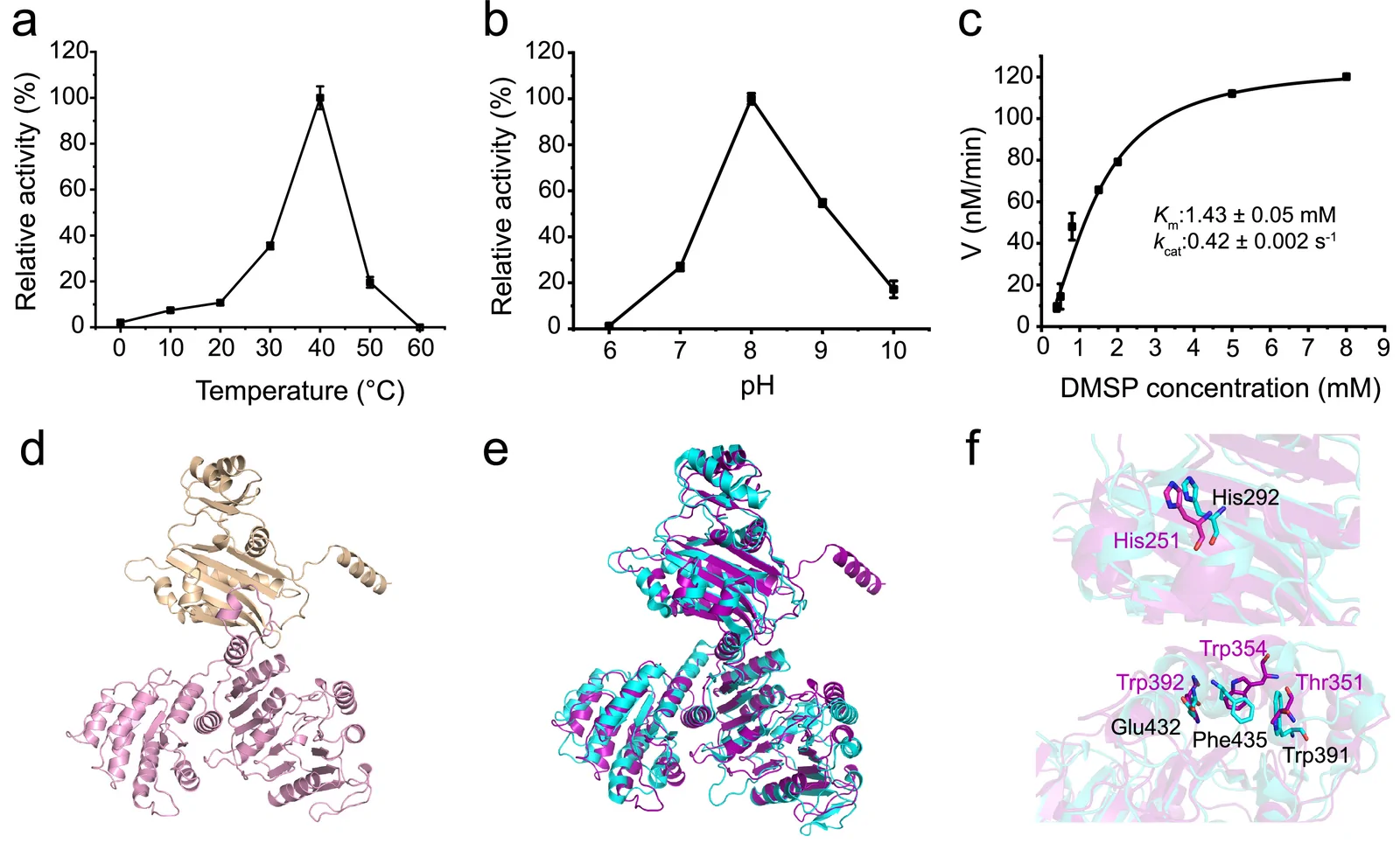
**Enzymatic characterization and structural prediction of the DddX-like enzyme.** (a) Effect of temperature on the enzymatic activity of the DddX-like enzyme. The error bars represent standard deviation of three independent biological replicates. (b) Effect of pH on the enzymatic activity of the DddX-like enzyme. The error bars represent standard deviation of three independent biological replicates. (c) Nonlinear fitting curves for DMSP cleavage by the DddX-like enzyme. The error bars represent standard deviation of three independent biological replicates. (d) The AlphaFold3-predicted structure of the DddX-like enzyme. (e) Structural alignment of the predicted DddX-like enzyme (purple) and DddX monomer (cyan, PDB ID: 7CM9). (f) Hypothetical key residues critical for substrate catalysis. Residues of the DddX-like enzyme were colored in purple, and those of DddX in cyan.

We further characterized the catalytic properties of the isolated DddX-like enzyme, and its optimal temperature and pH were found to be 40°C (Fig. 2a) and 8.0 (Fig. 2b), respectively, similar to those of DddX [33]. Notably, most reported DMSP lytic enzymes exhibit optimal temperatures well above those of their native marine habitats, as exemplified by DddX (40°C), DddP (60°C), DddY (60°C), DddK (50°C), and DddU (40°C) [33,[42], [43], [44],^29^]. Nevertheless, they retain substantial residual activities at 20–30°C, which are sufficient to support their physiological functions *in situ*. The recombinant DddX-like enzyme exhibited a *K*_m_ value of 1.43 mM and a *k*_cat_ value of 0.42 s^-1^ towards DMSP (Fig. 2c). Although the DddX-like enzyme exhibited a higher *K*_m_ value towards DMSP than DddX (0.4 mM), their binding affinities for DMSP fell into the same millimolar range [33]. In fact, many enzymes involved in bacterial DMSP catabolism usually possess relatively high *K*_m_ values at the millimolar level, including the DMSP lyases DddQ/K/W/L/Y/P/U and the DMSP demethylase DmdA [16]. This trait may facilitate the accumulation of DMSP in bacterial cells to realize its physiological roles, such as in stress protection [15,45].

To elucidate the catalytic mechanism of the DddX-like enzyme, we tried to determine its structure through crystallography or cryo-electron microscopy; however, these efforts all failed. We then predicted the structure of the DddX-like enzyme using AlphaFold3 (Fig. 2d) [46]. Despite their low amino acid sequence identities, the overall structure of the DddX-like enzyme was similar to that of DddX, with a root mean square deviation of 3.18 Å over 585 Cα atoms (Fig. 2e). Although the DddX-like enzyme maintained a dimeric structure in solution (Fig. S2), the monomer contained an intact active site (Fig. 2f). Thus, a monomeric comparison between the DddX-like enzyme and DddX was conducted. During the catalytic activity of DddX, it was expected that the residue His292 would be phosphorylated by ATP, residues Trp391 and Phe435 would participate in the binding of the intermediate DMSP-CoA, and Glu432 would act as the catalytic residue for the final cleavage of DMSP-CoA to produce DMS and acryloyl-CoA [33]. Structural alignment indicated that the residues His251, Thr351, Trp354, and Trp392 in the DddX-like enzyme corresponded to His292, Trp391, Phe435, and Glu432, respectively, in DddX (Fig. 2f). To explore the potential roles of these residues, we constructed four mutants (H251A, T351A, W354A and W392A), expressed and purified the recombinant proteins, and then measured their enzymatic activities. The results revealed that all these mutations significantly decreased the DMSP cleavage activity of the DddX-like enzyme (Fig. S4a), indicating their important roles in catalysis. CD spectroscopy analysis suggested that the secondary structures of the mutants exhibited little deviation from the WT DddX-like enzyme (Fig. S4b), indicating that the decrease in the enzymatic activities of the mutants resulted from amino acid substitutions rather than global structural changes.

Notably, although the mutational analysis suggested that residues Thr351 and Trp392 in the DddX-like enzyme participate in catalysis, their roles might differ from those of DddX. We expected that the residue Trp391 of DddX would bind to DMSP-CoA via the cation-π interaction formed between its aromatic side chain and the sulfonium group of the substrate [33]. However, the corresponding residue in the DddX-like enzyme, Thr351, does not possess a benzene ring in its side chain. Likewise, Glu432 of DddX was proposed to be the catalytic base that attacks the DMSP-CoA intermediate to release DMS and acryloyl-CoA [33]. However, it is unlikely that the corresponding hydrophobic residue Trp392 in the DddX-enzyme acts as a general base in this process. These results suggest that the DddX-like enzyme may employ a different catalytic mechanism, and this warrants further investigation.

### 3.3 Distribution of DddX and DddX-like enzymes

**Fig. 3:**
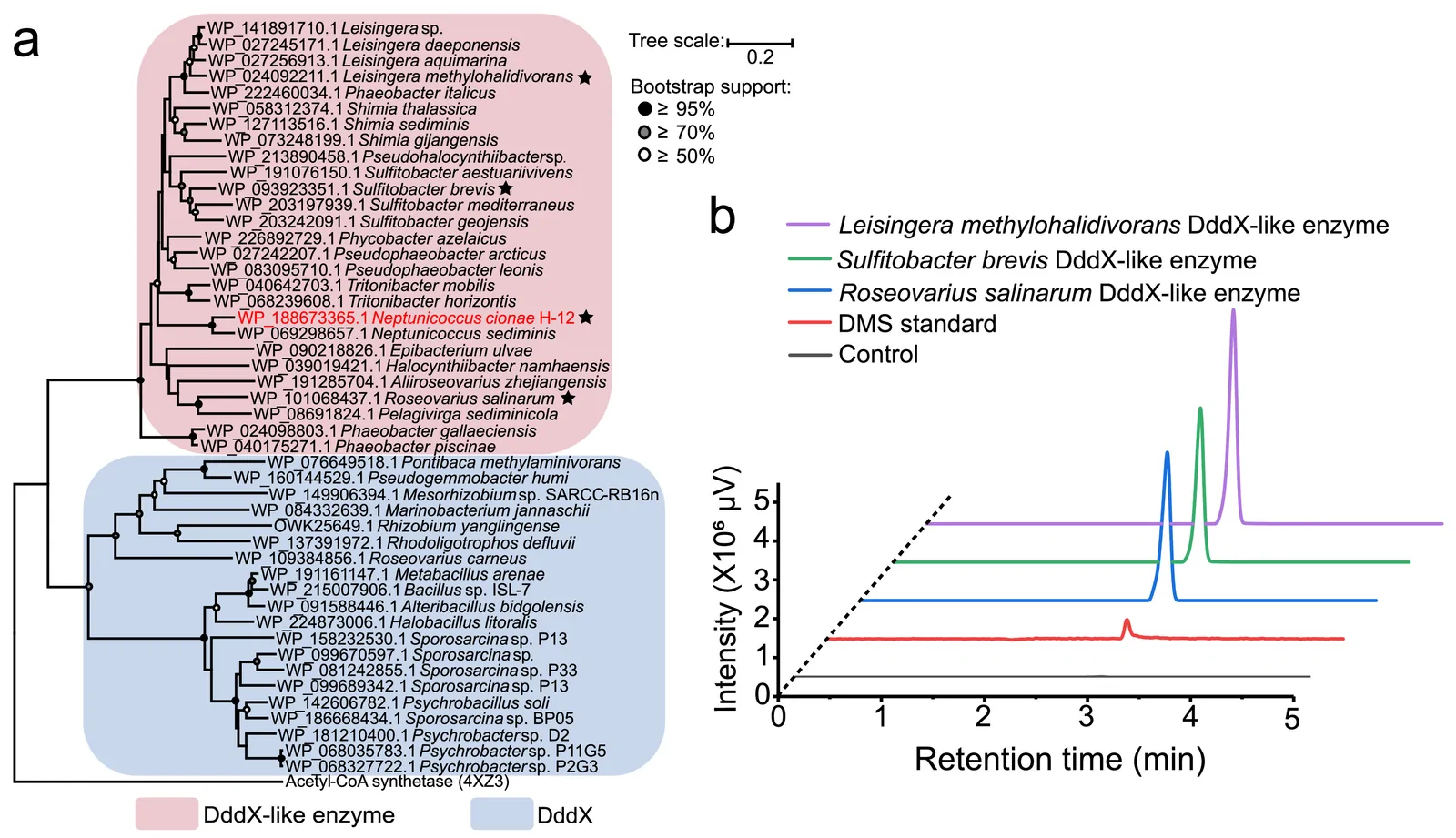
**Distribution of DddX-like enzyme in bacteria.** (a) The phylogenetic tree of DddX-like enzyme homologs and the reported DMSP lytic enzyme DddX. Those DddX-like enzyme homologs that were functionally characterized (WP_024092211.1 (*Leisingera methylohalidivorans*), WP_093923351.1 (*Sulfitobacter brevis*) and WP_101068437.1 (*Roseovarius salinarum*)) were marked with black pentagrams. Bootstrap values (≥50%) based on 1000 replicates were indicated at nodes. (b) GC detection of DMS produced from DMSP cleavage by DddX-like enzyme homologs.

To investigate the distribution of the DddX-like enzyme, a phylogenetic analysis was conducted using genome sequences available in the NCBI RefSeq database. The results suggested that the DddX-like enzyme homologs (>90% coverage and >60% amino acid sequence identity with the H-12 DddX-like enzyme) formed a separate clade from DddX (Fig. 3a). Unlike DddX, the DddX-like enzyme homologs were predominantly present in Alphaproteobacteria, particularly within the MRG (Fig. 3a). To validate the cleavage function of these DddX-like enzyme homologs, several representative *dddX*-like gene sequences from the MRG strains *Leisingera methylohalidivorans, Sulfitobacter brevis*, and *Roseovarius salinarum* were chemically synthesized, then expressed, and purified from *E. coli* BL21(DE3) cells. The results revealed that all the selected DddX-like enzyme homologs exhibited DMSP lytic activities (Fig. 3b), indicating that bacteria possessing this enzyme can catabolize DMSP.

**Fig. 4:**
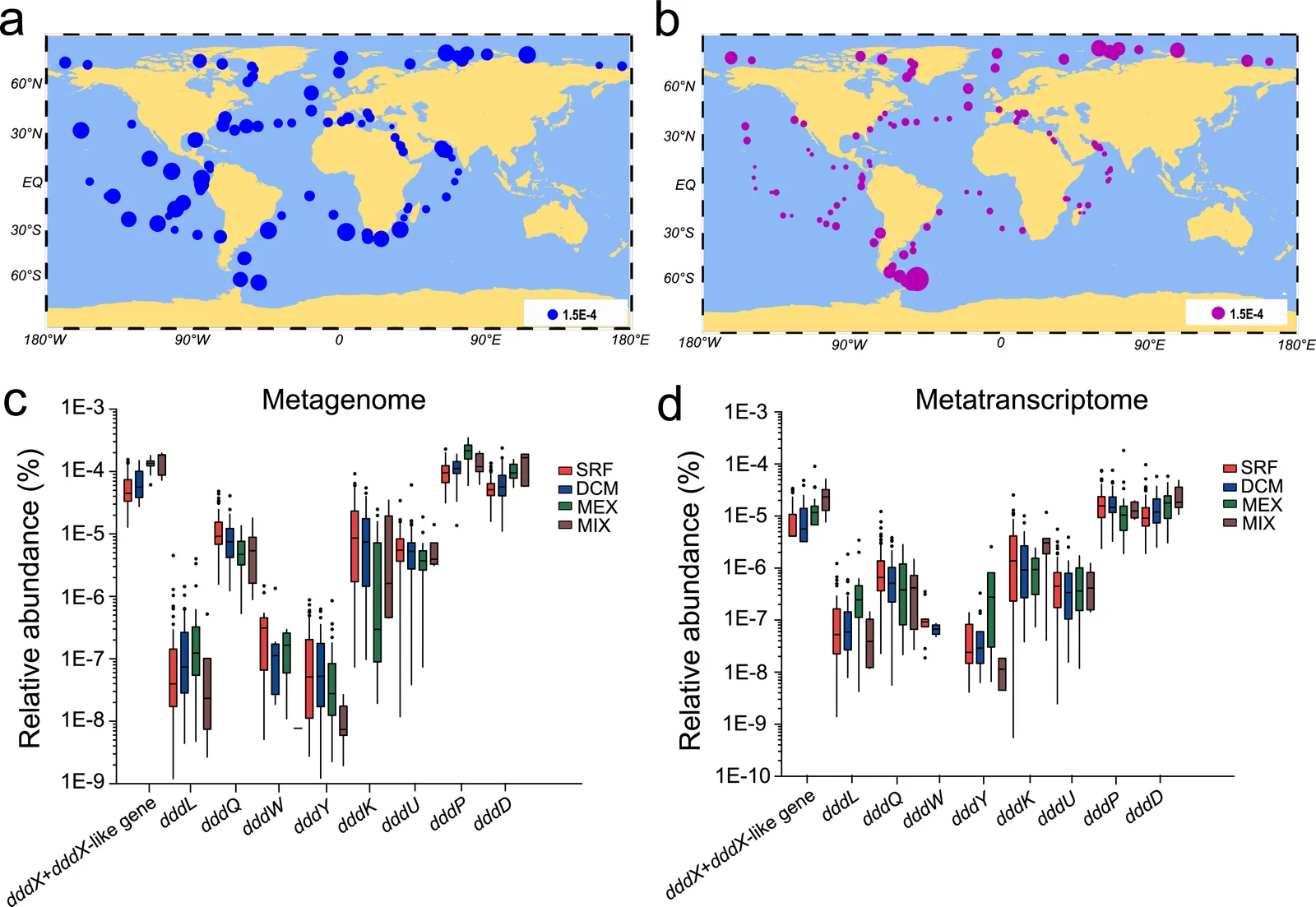
**Distribution of *dddX* and *dddX*-like genes in the *Tara Oceans* database.** (a) The geographic distribution of *dddX* and *dddX*-like genes. (b) The geographic distribution of *dddX* and *dddX*-like genes transcripts. (c) Normalized abundance of *dddX* and *dddX*-like genes and other reported bacterial DMSP-cleaving enzyme genes in *Tara Oceans* metagenomes at different sampling depths. (d) Normalized abundance of *dddX* and *dddX*-like genes and other reported bacterial DMSP-cleaving enzyme genes in *Tara Oceans* metatranscriptomes at different sampling depths. SRF, surface water layer. DCM, deep chlorophyll maximum layer; MES, mesopelagic layer; MIX, epipelagic wind mixed layer.

To evaluate the ecological significance of the *dddX*-like gene in marine environments, we analyzed its relative abundance in metagenomic and metatranscriptomic data from the *Tara Oceans* database [47]. Homology searches using HMMs with an e-value cut-off of ≤1e^-30^ retrieved a large number of shared homologs between *dddX* and *dddX*-like genes despite their phylogenetic divergence (Fig. 3a). Therefore, the *dddX* and *dddX*-like genes were analyzed together in subsequent distribution and abundance assessments. The *dddX* and *dddX*-like genes, along with their transcripts, were found to be broadly distributed across the global oceans, including the Atlantic, Pacific, Indian, and polar oceans (Fig. 4a, b). For reference, we also analyzed and compared the relative abundances of all reported bacterial DMSP lytic enzyme genes at different seawater depths. The results suggested that the abundance and transcriptional levels of *dddX* and *dddX*-like genes were remarkably high, comparable to those of the predominant genes *dddP* and *dddD*, and significantly higher than those of other DMSP lyase genes (e.g., *dddL, dddQ, dddW, dddY, dddK, dddU*) (Fig. 4c, d). These results suggest that the *dddX* and *dddX*-like genes play important roles in marine DMSP metabolism.

## 4 Conclusions

DMSP is an abundant organosulfur compound within marine environments, and its metabolite DMS plays a potential role in global climate regulation. In this study, we identified a functional DddX-like enzyme from the MRG strain *N. cionae* H-12, which catalyzes the cleavage of DMSP to generate DMS and acryloyl-CoA. Both DddX and DddX-like enzymes are prevalent in the oceans, indicating their important roles in global DMSP cycling. These findings provide deeper insights into the biochemical diversity of DMSP catabolism and expand our current understanding of the microbial processes driving global organic sulfur cycling.

## Data availability statement

The *dddX*-like gene sequence from strain *N. cionae* H-12 has been deposited in the NCBI RefSeq database with the accession number WP_188673365.1. The data supporting the findings of this study are available within the paper and its Supplementary Materials. Strains and plasmid constructs used in this study are available from the corresponding author upon reasonable request.

## CRediT authorship contribution statement

**Shu-Yan Wang:** Writing – original draft, Visualization, Formal analysis, Data curation. **Fei Xu:** Investigation, Formal analysis. **Meng Meng:** Formal analysis, Data curation. **Zeng-Tian Gu:** Software. **Min Zhang:** Validation, Formal analysis. **Yu-Zhong Zhang:** Supervision, Project administration, Funding acquisition. **Nan Zhang:** Funding acquisition. **Chun-Yang Li:** Writing – review & editing, Supervision, Funding acquisition, Conceptualization.

## Declaration of competing interest

The authors declare that they have no known competing financial interests or personal relationships that could have appeared to influence the work reported in this paper.

## Appendix Supplementary materials

Image, application 1
