# Supplementary material for: Identification of a functional DddX-like enzyme that catalyzes dimethylsulfoniopropionate cleavage in marine bacteria
Source: Eng Microbiol. 2026 Jul 30;6(4):100296. doi: 10.1016/j.engmic.2026.100296 (PMC13543886; doi:10.1016/j.engmic.2026.100296)
Supplement: Supplementary file 1 [file mmc1.docx]

**Supplementary materials**

Table S1. Strains and plasmids used in this study.

| Strain or plasmid | Description | Source or reference |
| --- | --- | --- |
| Strains  *Neptunicoccus cionae* H-12 | Wild-type isolate, previously named “*Amylibacter cionae* H-12” | CGMCC |
| Δ*dddX*-like gene | the *dddX*-like gene deletion mutant | This study |
| Δ*dddX*-like gene/pHG101-*dddX*-like gene | Δ*dddX*-like gene containing pHG101-*dddX*-like gene plasmid | This study |
| Δ*dddX*-like gene/pHG101 | Δ*dddX*-like gene containing pHG101 plasmid | This study |
| *Escherichia coli* WM3064 | Δ*dap*A, donor strain for conjugation | (Dehio and Meyer, 1997) |
| *Escherichia coli* DH 5α | Cloning strain | Vazyme Biotech company (China) |
| *Escherichia coli* BL21 (DE3) | Protein expression strain | Vazyme Biotech company (China) |
| Plasmids |  |  |
| pHGM01 | Gm^r^, *att*-based suicide vector | (Miao *et al.*, 2013) |
| pHGM01-Δ*dddX*-like gene | Gm^r^, pHGM01 containing the flanking region of *dddX*-like gene of *N. cionae* H-12 | This study |
| pHG101 | Km^r^, complementation vector without promoter | (Luo *et al.*, 2013) |
| pHG101-*dddX*-like gene | Km^r^, pHG101 containing the *dddX*-like gene of *N. cionae* H-12 | This study |
| pET-22b-*dddX*-like gene | Am^r^, protein expression vector containing the *dddX*-like gene of *N. cionae* H-12 | This study |

Gm^r^, gentamicin resistance. Km^r^, kanamycin resistance. Am^r^, [ampicillin](javascript:;) resistance.

Table S2. Primers used in this study.

| Primers | Sequence (5’→3’) |
| --- | --- |
| *dddX*-like gene-5O: | GGGGACAAGTTTGTACAAAAAAGCAGGCTCCCGAAGAAACGGATCACCA |
| *dddX*-like gene-5I | AAGTGCGCCTAATCGCGTAGATAAATCGCCCCCTGGAAGC |
| *dddX*-like gene-3I | CTACGCGATTAGGCGCACTTCAACCCGTTGATCTGTACGCC |
| *dddX*-like gene-3O | GGGGACCACTTTGTACAAGAAAGCTGGGTCGGTTATCGGCCTTTCCAGA |
| *dddX*-like gene-SF | TCCAAGGCGTCAGTTTCCAG |
| *dddX*-like gene-SR | TGATAGGGAATGCGCTTGGG |
| *dddX*-like gene-LF | GGGTTGTGAACGGGACCAA |
| *dddX*-like gene-LR  *dddX*-like gene*-*inF  *dddX*-like gene*-*inR | CAAATGGGCTTCTGCATCGT  GCGTCTTAATATCGCGCGGG  GCGGCAACAGCCTCTACTGT |
| *dddX*-like gene-pHG101-F | GCCCCGGGTGGTACCTGAATTCTCACGGACACAGCAATCCA |
| *dddX*-like gene-pHG101-R | GGTACTAGTAGGATCCCCTCGAGTCATGTCAGGGCCTTCTCG |
| H251A-F | TTATATCGCGATCATAATGCTGATGCGCCGGAACTGTAT |
| H251A-R | GCCAGCCAGTGACGCGGTGGCAGAAATGGTAGCTGCTTT |
| W392A-F | AAATGCTCTGATGCTGACGCGGATTGTGCGATCGACGCG |
| W392A-R | CGCGTCGATCGCACAATCCGCGTCAGCATCAGAGCATTT |

Fig. S1. The DMSP-dependent DMS production by the wild-type strain *Neptunicoccus cionae* H-12, Δ*dddU*, Δ*dddX*-like gene, and Δ*dddU*Δ*dddX*-like gene mutants. The error bars represent standard deviation of three independent biological replicates. *p < 0.05; ns, no significance (two-tailed Student's *t–*test).

Fig. S2. Gel filtration analysis of the DddX-like enzyme. Inset, semilog plot of the molecular mass of all standards used versus their *K*_av_ values (black dots). The red arrow indicates the position of the *K*_av_ value of the DddX-like enzyme interpolated in the regression line. The predicted molecular mass of the DddX-like enzyme is 71.63 kDa.

Fig. S3. Cofactor dependence analysis of the DddX-like enzyme activity.

Fig. S4. Mutational analysis of the DddX-like enzyme. (a). DMSP lyase activity of the DddX-like enzyme and its amino acid substitution mutants. The activity of the wide-type DddX-like enzyme was defined as 100%. The error bars represent standard deviation of three independent biological replicates. **p < 0.01, ***p < 0.001 (two-tailed Student's *t*–test). (b). CD spectra of the wide-type DddX-like enzyme and its mutants H251A, T351A, W354A and W392A. Curves represent the mean of three technical replicates.
